# Incidence and Effects of Polypharmacy on Clinical Outcome among Patients Aged 80^+^: A Five-Year Follow-Up Study

**DOI:** 10.1371/journal.pone.0142123

**Published:** 2015-11-10

**Authors:** Rong Wang, Lei Chen, Li Fan, Dewei Gao, Zhiru Liang, Jing He, Weiqin Gong, Linggen Gao

**Affiliations:** 1 Department of Comprehensive Surgery, General Hospital of Chinese People's Liberation Army, Beijing, China; 2 Department of Thoracic Surgery, General Hospital of Chinese People's Liberation Army, Beijing, China; 3 Department of Geriatric Cardiology, General Hospital of Chinese People's Liberation Army, Beijing, China; Baylor College of Medicine, UNITED STATES

## Abstract

**Objectives:**

Polypharmacy is a problem of growing interest in geriatrics with the increase in drug consumption in recent years, is defined according to the WHO criteria as the, ‘‘concurrent use of five or more different prescription medication”. We investigated the clinical characteristics of polypharmacy and identified the effects of polypharmacy on clinical outcome among patients aged 80+ admitted to Chinese PLA general hospital.

**Methods:**

Older men aged ≥80 years (n = 1562) were included in this study. The included participants attended a structured clinical examination and an interview carried out by a geriatrician and trained nurses. A follow-up survey in 2014 was carried out on survivors in the same way as in 2009. The clinical outcome measured were adverse drug reactions, falls, frailty, disability, cognitive impairment, mortality. The association between polypharmacy and clinical outcome was assessed by logistic regression.

**Results:**

The mean (range) age of the included participants was 85.2 (80–104) years. Medication exposure was reported by 100% of the population. Mean number of medications reported in this population was 9.56±5.68. The prevalence of polypharmacy (≥6 medications) in the present study was 70%. At the time of the follow-up survey, an increase in the number of taken medicines had occurred among half of the survivors. The risk of different outcomes in relation to number of medications rises significantly, the odds ratios were 1.21 (95% confidence interval [CI]1.17–1.28) for adverse drug reactions, 1.18 (95% CI 1.10–1.26) for falls, 1.16 (95% CI 1.09–1.24) for disability, and 1.19 (95% CI 1.12–1.23) for mortality. There was no association between increasing number of medications and cognitive impairment.

**Conclusions:**

Our study demonstrates that polypharmacy is very common in the very old patients, and observed that number of medications was a factor associated with difference clinical outcome independently of the age, type of medications prescribed and accompanied comorbidities.

## Introduction

Population ageing is an increasing worldwide phenomenon [1]that means higher demands on health care, including the use of medications. The older population suffer from chronic diseases and multimorbidity[2–4] and are treated with an increasing number of drugs. Along with age-related changes in pharmacokinetics and pharmacodynamics, polypharmacy becomes a problem with negative clinical consequences and a resulting increase in the economic costs of healthcare. A systematic review published in 2013 noted that polypharmacy has a clearly established strong relationship with negative clinical outcomes [5]. Several previous studies have also reported that polypharmacy is associated with the increased occurrence of inappropriate medication, drug-drug interactions, adverse drug reactions[6–8], and poorer health outcomes such as functional impairment, malnutrition, falls, fractures, and hospitalization [9–13].

Although there is an evidence on the association of polypharmacy with adverse outcomes in older adults, studies examining polypharmacy in oldest old patients in China are limited. The objective of the present study is to identify effects of polypharmacy on clinical outcome among patients aged 80^+^ admitted to our hospital.

## Methods

### Data Collection

Patients were recruited at the geriatric outpatient clinic on the occasion of routine check-up visits in the South Building of Chinese PLA General Hospital in 2009. All participants in this study were the leaders of Chinese People's Liberation Army, had been provided VIP health care services including individualized health exam and medical healthcare programs by high-quality specialists and currently in a stable clinical status. This study excluded patients with advanced disease (cancer or noncancer) in whom the initial estimate of life expectancy was less than 3 months and patients in whom follow-up availability was shorter than 3 months. Subjects who were transferred to inpatient departments directly from clinic were not recruited. If the participant was unable to answer the questions, a close relative or a friend could give the required information. The included participants attended a structured clinical examination and an interview carried out by a geriatrician and trained nurses. A follow-up survey in 2014 was carried out on survivors in the same way as in 2009. Patients were interviewed using a questionnaire that included medical histories, current diagnoses and drug use were recorded from a combination of electronic and paper-based records. Data on medication use was extracted from the medication management plan, a form used by clinical pharmacists to document patients’ medication use prior to and during admission. Drug use refers to regular and as-needed consumption of regularly and as-needed taken drugs, vitamins and mineral supplements. Drugs taken daily or at regular intervals were defined as being in regular use. Whereas occasionally taken drugs were defined as as-needed taken drugs. Polypharmacy status was defined as a three-class variable. Excessive polypharmacy was defined as the use of ten or more drugs, polypharmacy as the use of six to nine drugs, and non-polypharmacy as the use of five or less drugs concomitantly.

### Outcome measures

#### Adverse drug reactions

We obtained information on adverse drug reactions(ADR) defined as ‘‘an appreciably harmful or unpleasant reaction, resulting from an intervention related to the use of a medicinal product, which predicts hazard from future administration and warrants prevention or specific treatment, or alteration of the dosage regimen, or withdrawal of the product.”[14]. A reporting system of ADR was introduced in the South Building of Chinese PLA General Hospital. The patients were phoned every 3 months and asked whether they had developed ADR. If someone thought they might have a problem that was a ADR they could come to the geriatric outpatient clinic. Patients were interviewed about signs and symptoms related to their drug therapy. Patient medical records were also reviewed for collection of data. Two physicians and one pharmacist independently reviewed each reported ADR to determine the likelihood that the event was connected to a medication. A thorough literature search was performed before labeling any case as an ADR[15]. Besides this, the standard ADR Reporting Form was also used to record all the essential information regarding the adverse effects: suspected drugs, suspected reaction, date of onset, date when the adverse effects ceased and severity of the ADR experienced. Subjects with ADR were formally referred to their primary investigators.

#### Falls

Polypharmacy contributes to falls and hip fractures[16],which are associated with high morbidity and mortality rates in the elderly[17].Participants were phoned every 3 months and asked the following question ‘‘Have you fallen in the past 3 months?” Participants were classified as having two or more falls compared with one or none.

#### Frailty

Frailty is a syndrome of decreased physiologic reserve and resistance to stressors. Fried et al. proposed a widely-recognized criteria, which has been validated for elderly patients[18–20].Participants were considered frail when they had at least three of these five criteria: exhaustion, weakness, low physical activity, slow walking speed, and weight loss, defined as follows. Exhaustion: an affirmative answer to either of the following two questions, “I felt that anything I did was a big effort” and “I felt that I could not get going,” with a frequency higher than 3 to 4 days per week. Weakness: the lowest quintile in the Cardiovascular Health Study of maximum strength on the dominant hand adjusted for body mass index (BMI). Results were based on the highest value of two strength measures using a Jamar dynamometer. ((BMI <22.1 kg/m^2^, strength1 kg/med on the ^2^≤ strength1 kg^2^, strength1 kg/med on the hig^2^≤ strength1 kg/^2^ strengthh1 kg/m; BMIrengthh1 k^2^, strength1 kg/med) Low physical activity: walking 2.5 hours per week or less in men and 2 hours per week or less in women. Slow walking speed: the lowest quintile in the study sample for the 3-m walking speed test adjusted for sex and height. Weight loss: self-reported involuntary loss 4.5 kg or more of body weight in the last year. The patients were examined and asked whether they had at least three of these five criteria. Medication data were collected by completing the questionnaire, which were distributed to outpatient clinics.

#### Disability

Disability was assessed using the activities of daily living (ADL), self-reported scale at baseline [21]. Disability in ADL was defined as needing help with one or more activities included in ADL scale (walking, bathing, personal grooming, dressing, eating, getting from bed to chair, and using the toilet). Participants were classified as disabled or not.

#### Cognitive capacity

Cognitive capacity was measured by the minimental state examination (MMSE) screening test. This 30-point questionnaire samples various functions of cognition, including arithmetic, memory and orientation. The maximum scores are 30, meaning good cognitive capacity, whereas 24 or below meaning impaired capacity. In this study, cognitive impairment was coded as absent if the subject scores over 24 on the MMSE screening test and present if the score was 24 or less.

#### Mortality

All men were phoned at three monthly intervals from the baseline clinic assessment, which enabled regular updating of survival data. Data on deaths based on the death registry records were available from the clinical database.

## Statistical Analysis

The distributions of baseline descriptive statistics across polypharmacy groups are expressed as proportions and means with standard deviations (SDs).In the case of categorical variables, cross-tabulations with chi-square tests were used in comparing the differences between polypharmacy groups. For continuous variables, the statistically significant difference in means between polypharmacy groups was determined by the analysis of variance (ANOVA).The association between polypharmacy and clinical outcome was assessed by logistic regression. We adjusted for age, type of medications and comorbidities. The results are shown as odds ratios (ORs) with 95% CIs.

Statistical Package for Social Sciences (SPSS) Version 19 was used for analysis. The results were considered as statistically significant at level p<0.05.

## Ethics Statement

Written informed consent was obtained from the subjects or their relatives. The study protocol was approved by the Ethics Committee of General Hospital of Chinese PLA, Beijing, China.

## Results

A total of 1562 elderly people aged ≥80 years from the Chinese PLA general hospital in 2009 were included in this study. Characteristics of the study population are described in Table 1. The mean (range) age of the men in the study population was 85.2 (80–104) years. All participants in this study using some medication. Mean number of medications in this population was 9.56±5.68.

The prevalence of the most common conditions were hypertension 52.1%, ischemic heart disease 33.5%, diabetes 24.5%, pain 28.7%, chronic kidney disease 16.8%. Benign prostate hypertrophy, stroke, constipation, dyslipidemia and hearing loss made up the rest of the top 10 most prevalent conditions.

**Table 1:** Baseline characteristics of the study population (n = 1562).

Characteristics	Value
Age, mean(Range) ys	85.2(80–104)
Number of drugs prescribed mean(SD)	9.56 ±5.68
Number of prescriptions per patients	
<5	469
5–10	532
11–20	447
>20	114
Range of drug prescriptions per patient	2–26
Number of comorbidities mean(SD)	4.3(3.7)
Type of comorbidity	
Hypertension N(%)	813(52.1)
Ischaemic heart disease N(%)	523(33.5)
Diabetes N(%)	382(24.5)
Pain N(%)	448(28.7)
Chronic kidney disease N(%)	262(16.8)
Frail status N(%)	295(18.9)
Disability on ADL N(%)	226(14.5)
Cognitively impaired N(%)	202(12.9)

The top seven most frequently used classes of medications were anti-hypertensives, antiplatelet agents, hypoglycemic agents, analgesics, gastrointestinal agents, purgatives, anti-hyperlipidaemics. The prevalence of polypharmacy among the participants was 70%. A total of 295 (18.9%) participants were identified as being frail. Disability on activities of daily living was observed in 226(14.5%) of participants, and 202(12.9%) were cognitively impaired. The mortality rate was 5.06% during a mean follow-up period of 60.2months. Falls were reported by (556) 35.6% of participants.

Among the survivors, the average number of medicines in use increased from 8.15 to 9.17(p<0.001) in the polypharmacy group and from 16.39 to 17.28 in the excessive polypharmacy group during the follow-up (Table 2). At the time of the follow-up survey, an increase in the number of taken medicines had occurred among half of the survivors (50.9%, n = 921).

**Table 2:** Changes (%) in functional ability (IADL test) and cognitive capacity (MMSE test) among survivors from 2009 to 2014.

Characteristics	2009	2009	2009	p-value	2014	2014	2014	p-value
	NP	P	EP		NP	P	EP	
	N = 469	N = 532	N = 561		N = 396	N = 521	N = 581	
Age, mean(SD) ys	84.1(4.5)	83.9(4.0)	84.5(4.8)	0.599	88.9(4.2)	88.6(4.9)	89.7(4.6)	0.006
Number of medications mean(SD)	3.23(1.21)	8.15(2.48)	16.39(6.97)	<0.001	3.79(1.98)	9.87(3.05)	17.28(7.02)	<0.001
Number of comorbidities mean(SD)	2.5(1.2)	3.8(1.9)	6.6(2.4)	<0.001	2.9(1.5)	4.2(1.8)	6.9(2.6)	<0.001
ADR(n)	57	74	102	<0.05	62	88	115	<0.05
Falls(n)	86	108	131	<0.05	92	120	140	<0.05
Frail(n)	143	156	166	0.261	169	178	196	<0.05
Disability(n)	51	64	82	<0.05	59	86	98	<0.05
Cognitive capacity(n)	66	72	68	0.578	79	88	97	0.652

NP, Non-polypharmacy; P, Polypharmacy; EP, Excessive Polypharmacy; ADR, Adverse Drug Reactions. p-Values for categorical variables were measured with a chi-square (x^2^) test and for continuous variables with ANOVA.

The risk of different outcomes in relation to number of medications rises significantly (Table 3), the odds ratios were 1.21 (95% confidence interval [CI] 1.17–1.28) for adverse drug reactions, 1.18 (95% CI 1.10–1.26) for falls, 1.16 (95% CI 1.09–1.24) for disability, and 1.19 (95% CI 1.12–1.23) for mortality. There was no association between increasing number of medications and cognitive impairment [22].

**Table 3:** ORs of different outcomes in relation to number of medications.

Outcomes	Unadjusted OR (95% CI)	P-value	Age adjusted OR (95% CI)	P-value	*Adjusted OR(95% CI)	P-value
ADR	1.21(1.17,1.28)	<0.0001	1.25(1.13,1.33)	<0.0001	1.09(1.02,1.15)	<0.0001
Falls	1.18(1.10,1.26)	<0.0001	1.15(1.13,1.21)	<0.0001	1.08(1.01,1.16)	<0.0001
Frailty	1.29(1.21,1.37)	<0.0001	1.23(1.18,1.32)	<0.0001	1.06(1.02,1.11)	0.003
Disability	1.16(1.09,1.24)	<0.0001	1.13(1.08,1.18)	<0.0001	1.04(1.02,1.15)	<0.0001
Cognitive capacity	0.87(0.81,1.09)	0.56	0.95(0.90,1.02)	0.42	0.98(0.92,1.05)	0.55
Mortality	1.19(1.12,1.23)	<0.0001	1.14(1.06,1.19)	<0.0001	1.06(1.00,1.15)	0.0009

ADR, Adverse Drug Reactions; CI,Confidence interval; OR, odds ratio. ORs are for risk with increasing of medications by one.

*Adjusted for age, type of medications and comorbidities.

## Discussion

The present prospective study confirmed that the number of medicines and the prevalence of polypharmacy and excessive polypharmacy increases with advancing age. And polypharmacy increased the different risk of clinical outcomes during the three-year period for those elderly persons. These findings are consistent with previous studies reporting polypharmacy is associated with adverse drug reactions, falls and functional disability in elderly persons [23–25]. This further supports the interpretation that polypharmacy may act as an indicator of overall worsening health.

In the present study, the prevalence of polypharmacy among the participants was 70%. Why is polypharmacy so widespread? The main reason is that polypharmacy and excessive polypharmacy occur mostly because of increased morbidity with aging. Physicians tend to follow the guidelines derived from clinical trials that have not included frail older people or those with multiple morbidities and prescribe all the drugs recommended for each disease that affect the elderly. Hence, guidelines are needed to take into account multimorbidity and polypharmacy. It is necessary to assess the medication regimen at regular intervals. This presents a challenge caring for older people.

The risk of different outcomes in relation to number of medications rises significantly in our study. The odds ratios were 1.21 (95% CI 1.17–1.28) for adverse drug reactions and common drug classes associated with adverse drug reactions included antihypertensives, antiplatelet agents, hypoglycemic agents, antihyperlipidaemics. A population based study demonstrated that patients taking five or more medications had an 88% increased risk of experiencing adverse drug reactions compared to those who were taking fewer medications [26]. Given the heterogeneity within the older population, providing individualized care is pivotal to preventing adverse drug reactions. Polypharmacy has been associated with functional decline in older patients. In the present study, the risk of falls in relation to number of medications rises significantly, the odds ratios was 1.18 (95% CI 1.10–1.26). This result is consistent with previous studies reporting that the number of medications was associated with an increased risk of falls[27–31].

In the present study we also found that multiple medications also contribute to excessive mortality in old people, the odds ratios was 1.19(1.12,1.23). Espino et al. reported an increased risk of death associated with polypharmacy (HR 1.27, 95% CI 1.04–1.56) in a cohort study with an 8-year follow-up period [32]. However, Jyrkkä and colleagues (polypharmacy HR 0.77, 95% CI 0.58–1.01)[33] and Pozzi and colleagues (HR 1.20, 95% CI 0.89–1.60)[34]reported no such association.

## Limitations

This study is subject to certain limitations. The sample of patients came from a single health centre and all of them were male.

## Conclusion

The major strength of this study is the large number of patients 80 years of age and older who were included. Our study clearly demonstrates that polypharmacy is very common in the most multimorbid patients, and observed that number of medications was a factor associated with difference clinical outcome independently of the age, type of medications prescribed and accompanied comorbidities. Well-designed intervention studies that focus on enrolling high risk older patients with polypharmacy have shown that they can be effective in improving the overall quality of prescribing with mixed results on distal health outcomes.
